# Computational methods applied to syphilis: where are we, and where are we going?

**DOI:** 10.3389/fpubh.2023.1201725

**Published:** 2023-08-23

**Authors:** Gabriela Albuquerque, Felipe Fernandes, Ingridy M. P. Barbalho, Daniele M. S. Barros, Philippi S. G. Morais, Antônio H. F. Morais, Marquiony M. Santos, Leonardo J. Galvão-Lima, Ana Isabela L. Sales-Moioli, João Paulo Q. Santos, Paulo Gil, Jorge Henriques, César Teixeira, Thaisa Santos Lima, Karilany D. Coutinho, Talita K. B. Pinto, Ricardo A. M. Valentim

**Affiliations:** ^1^Laboratory of Technological Innovation in Health, Federal University of Rio Grande do Norte, Natal, Rio Grande do Norte, Brazil; ^2^Advanced Nucleus of Technological Innovation (NAVI), Federal Institute of Rio Grande do Norte, Natal, Rio Grande do Norte, Brazil; ^3^Department of Informatics Engineering, Center for Informatics and Systems of the University of Coimbra, Universidade de Coimbra, Coimbra, Portugal; ^4^Ministry of Health, Esplanada dos Ministérios, Brasília, Brazil

**Keywords:** public health, digital health, intelligent systems, artificial intelligence, machine learning

## Abstract

Syphilis is an infectious disease that can be diagnosed and treated cheaply. Despite being a curable condition, the syphilis rate is increasing worldwide. In this sense, computational methods can analyze data and assist managers in formulating new public policies for preventing and controlling sexually transmitted infections (STIs). Computational techniques can integrate knowledge from experiences and, through an inference mechanism, apply conditions to a database that seeks to explain data behavior. This systematic review analyzed studies that use computational methods to establish or improve syphilis-related aspects. Our review shows the usefulness of computational tools to promote the overall understanding of syphilis, a global problem, to guide public policy and practice, to target better public health interventions such as surveillance and prevention, health service delivery, and the optimal use of diagnostic tools. The review was conducted according to PRISMA 2020 Statement and used several quality criteria to include studies. The publications chosen to compose this review were gathered from Science Direct, Web of Science, Springer, Scopus, ACM Digital Library, and PubMed databases. Then, studies published between 2015 and 2022 were selected. The review identified 1,991 studies. After applying inclusion, exclusion, and study quality assessment criteria, 26 primary studies were included in the final analysis. The results show different computational approaches, including countless Machine Learning algorithmic models, and three sub-areas of application in the context of syphilis: surveillance (61.54%), diagnosis (34.62%), and health policy evaluation (3.85%). These computational approaches are promising and capable of being tools to support syphilis control and surveillance actions.

## 1. Introduction

Syphilis is an infectious disease caused by *Treponema pallidum* subsp. *pallidum* (*T. Pallidum*) infection that can be sexually transmitted (Acquired Syphilis—AS) or through vertical transmission during pregnancy (Congenital Syphilis—CS) (1–3). Although curable and preventable through barrier methods (such as condoms), syphilis has been neglected and still represents a global public health concern due to inadequate diagnosis and treatment, resulting in morbidity and mortality in newborns and untreated infected people (4, 5).

According to the World Health Organization (WHO), over 357 million new cases of curable sexually transmitted infections (STIs) were diagnosed among young adults (15–49 years) in 2016 alone, of which 6 million were associated with syphilis (6). Currently, Brazil, Europe Union, and the USA are facing a silent syphilis epidemic that affects millions of patients annually (7–10). The 2022 Epidemiological Bulletin of Syphilis reported that, between January 1 and June 30, 2021, 167,523 new cases of AS were identified, followed by 74,095 cases of syphilis in pregnancy (SIP) and 27,019 cases of CS (11). In Brazil, there was on average one new case of AS every 1 min and 40 s, 1 new case of SIP every 4 min and 15 s, and 1 new case of CS every 11 min (12, 13).

Syphilis is diagnosed through serological tests, such as the Venereal Disease Research Laboratory (VDRL), a non-treponemal test. If a non-treponemal test is reactive, a treponemal test, e.g., *T. Pallidum* hemagglutination assay (TPHA), is performed to confirm the diagnosis. However, serological tests have limitations, such as the time of infection, which may present false-negative results in cases of early or late infections (14).

Adequately treated patients are expected to show significantly reducing non-treponemal antibody titers, but there are cases where titers persist for months to years and may represent a false-positive result when retested (15, 16). Furthermore, it is also possible to observe false-positive results in patients with an autoimmune condition, such as systemic lupus erythematosus, with other infectious diseases, such as brucellosis, or even in pregnancy (15). As syphilis shares several clinical manifestations and clinical characteristics with other treponemal and non-treponemal diseases, a safe clinical diagnosis is necessary, always performed by well-prepared and highly accurate laboratory tests (17).

In parallel, computational methods have been applied in health to aid diagnosis and treatment decisions, including in the diagnosis of STIs, recommendation of adequate treatment, and predictions on the probability of infection (18–21). Predictive analytics is a method for predicting future risks based on current and prior data, assisted often by data mining, machine learning, and novel statistical techniques (22). These techniques are used to develop an inference mechanism, a set of rules that can be applied to a dataset to render a mathematical function that can predict or infer knowledge about that data (19).

Artificial intelligence (AI) has been used to determine characteristics of individuals who are more prone to STIs, such as men who have sex with men (MSM), transgender people, sex workers, those who use stimulants to enhance and prolong sexual experiences (known as chemsex practitioners), and pre-exposure prophylaxis users (PrEP) who do not use condoms (23). For AI systems to be deployed, they need to be trained using data generated from clinical interactions. These data can be collected during clinical activities such as screening, diagnosis, and treatment of patients so that the AI systems can learn the similarities between groups and associations between the characteristics of subjects. This data can also include demographic data, clinical notes of health professionals, electronic records from medical devices, data from physical exams, and laboratory and imaging results. AI includes, among others, machine learning (ML) techniques that analyze structured data, such as images and genetic data, and natural language processing (NLP) that can use and integrate data in various forms, such as text, waveform, and images (24).

Basic ML algorithms can be categorized as supervised and unsupervised. Supervised ML methods work by gathering many training cases, which contain labeled inputs and the desired outputs (25). By analyzing the patterns in all the labeled input-output pairs for new cases, the algorithm learns how to produce the correct output for a given input (26, 27). Unsupervised learning infers the underlying patterns by applying similarity measures to unlabeled data to find subclusters of the original data, identify outliers, or produce low-dimensional representations of the data (24).

Against this background, this systematic literature review (SLR) aims to analyze published studies that use computational methods with the application of AI, ML, or other statistical methods to predict the occurrence of syphilis in critical populations and also identify potential gaps and opportunities for future research on different areas for programmatic response to syphilis, such as management of surveillance and comprehensive care.

## 2. Materials and methods

This research was developed based on the systematic review guidelines proposed by Kitchenham (28) and the PRISMA checklist (29). Initially, as a fundamental part of the protocol, 3 Research Questions (RQ) were formulated (Table 1).

**Table 1:** Research questions.

**RQ**	**Description**
01	What computational methods are being applied to syphilis?
02	What is the purpose of applying computational methods in the context of syphilis?
03	In which areas of health are computational methods being applied (surveillance, diagnosis/prediction, or evaluation of public health policies)?

The process of identifying primary studies related to the research object of this SLR consisted of searches in six repositories: Science Direct, Web of Science, Springer, Scopus, ACM Digital Library, and PubMed. Searches in all databases were performed on August 9, 2022. The following search string (SS01) was used in searches:

- (syphilis) AND (“machine learning” OR “artificial intelligence” OR “computational intelligence” OR “deep learning” OR fuzzy OR “artificial neural network” OR “specialist systems” OR “smart system”).

After identifying and defining the initial set of records, screening was performed to select a subset of eligible primary studies. This process was organized and executed based on the application of three basic procedures: (i) Inclusion Criteria—IC; (ii) Exclusion Criteria—EC; and (iii) Quality Assessment Criteria—QA.

In the first procedure (i), a subset of primary studies was defined from the IC and applied through the filters available in the repositories. In the subsequent step (ii), a screening guided by the EC based on reading the title, abstract, and keywords was performed on the subset of primary studies. Rayyan (30), a web application for systematic reviews, helped carry out step (ii). The search used two inclusion and three exclusion criteria, as shown in Table 2.

**Table 2:** Inclusion and exclusion criteria.

***N***	**Inclusion criteria**	**Exclusion criteria**
01	Articles published from 2015 to 2022	Duplicate articles
02	Research articles	Review articles
03	–	Studies not related to syphilis and computational methods

To determine the final set of eligible studies to seek answers to the RQ (Table 1), a screening guided by the QA criteria was carried out from the complete reading of the primary articles (Table 3). An evaluation metric called *score* was used to qualify and classify the studies (as presented in Equation 1). The *score* is the arithmetic mean of the weights (*w*) assigned to each QA criterion. The weight (*w*), which can vary between 0, 0.5, and 1.0, measures how satisfactory the response of that article is to a specific QA criterion, as shown in Equation (2). The preliminary reports that obtained a *score* ≥ 0.5 (i.e., 0.5 ≤ *score* ≤ 1.0) were considered eligible for this SLR.


(1) $$\begin{array}{l} score=\frac{1}{n_{\text{QA}}}\overset{n_{\text{QA}}}{\underset{i=1}{\sum }}w_{{\text{QA}}_{i}} \end{array}$$

where:

– *n*_*QA*_: variable used to represent the total of QA criteria;
– *w*_*QA*_: variable used to determine the value referring to the weight w attributed to the QA criterion under analysis (see the possible values in Equation 2).

(2) $$\begin{array}{l} w_{\text{QA}}=\{\begin{array}{ll} 1.0, & \text{yes},\text{fully describes},\\ 0.5, & \text{yes},\text{partially describes},\\ 0, & \text{does not describe}. \end{array} \end{array}$$

**Table 3:** Quality assessment.

**QA**	**Description**
01	Does the study have as an object of investigation a computational approach applied to the topic of syphilis?
02	Does the study describe the computational method applied to the context of syphilis?
03	Does the study describe the field of application in health (surveillance, diagnosis, and evaluation of public policies)?

**Figure 1:**
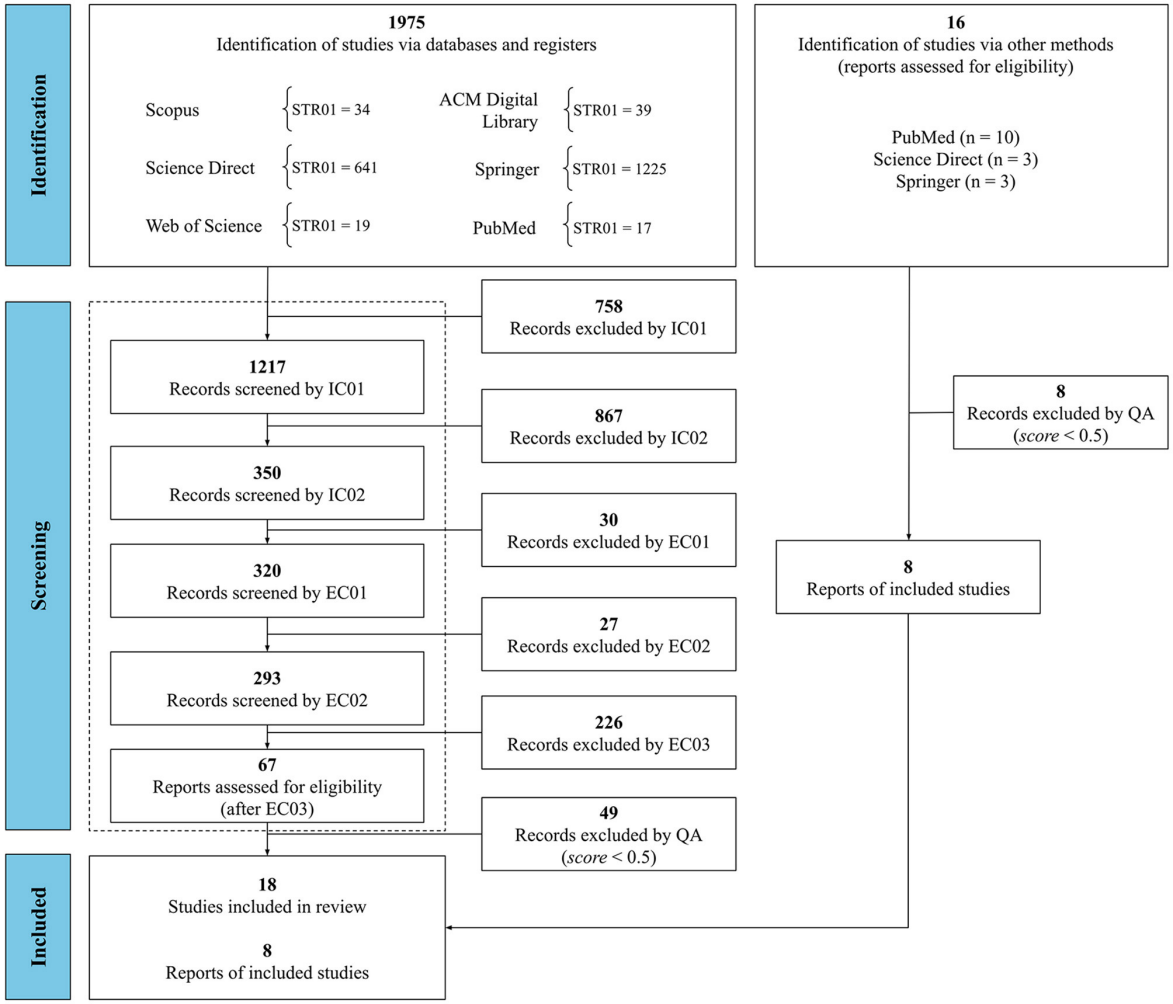
Adapted from PRISMA 2020 flow diagram from the result of the execution of the systematic review protocol.

The *scores* were assigned by two independent reviewers and elementary data of the final set of eligible studies, extracted based on the RQ, were summarized in Table 4. Studies were included via another method, based on a simple and active search in Science Direct, Springer, and PubMed (Figure 1). This search used the following descriptors: syphilis AND model AND diagnosis.

**Table 4:** Set of selected primary studies and their main characteristics.

**References**	**Year**	**Score**	**Health target**	**Objective**	**Techniques (best model)**	**Performance (best model)**			

						**Acc**	**Recall**	**Precision**	**AUC**
Xu et al. (31)	2022	1.0	Surveillance	Predicting risk	Boosted GLM	–	–	–	76%
Valentim et al. (32)	2022	1.0	Surveillance	Predicting syphilis	Stochastic Petri net and three regressions	98.81%	–	–	–
Yan et al. (33)	2021	1.0	Surveillance	Predicting STDs	ARIMA	**RMSE**: 1,794.99; **MAPE**: 3.39%			
Cuffe et al. (34)	2020	1.0	Surveillance	Predicting syphilis	Logistic Regression	–	88.1%	–	89.2%
Young et al. (35)	2018	1.0	Surveillance	Predicting syphilis	LMM and LASSO	**RMSE**: 4.90; **R**^**2**^: 0.898			
Allan-Blitz et al. (36)	2018	1.0	Surveillance	Predicting syphilis	GEE and Poisson Regression	–	–	–	69%
Macedo et al. (37)	2016	1.0	Surveillance	Recommending information	CISS+ (NLP)	–	–	90%	–
Zhang et al. (38)	2016	1.0	Surveillance	Estimating syphilis incidence	ARIMAX	RMSE^*^: 0.0097; MAPE^*^: 0.1335			
Yan et al. (39)	2022	0.83	Surveillance	Analyzing the impact of the COVID-19 pandemic on the epidemiological changes of STDs	Gray Model	**APE 2019/2020**: 4.07%/15.45%			
Tissot and Pedebos (40)	2021	0.83	Surveillance	Assessing clinical risk	KER	**AUPRC**: 0.099			
Joshi et al. (41)	2021	0.83	Surveillance	Estimating syphilis cases	ARIMA	–	–	–	–
Amith et al. (42)	2020	0.83	Surveillance	Analyzing social networks	Ontology	***z*****-score**: 0.48			
Serban et al. (43)	2019	0.83	Surveillance	Forecasting outbreaks/levels of disease	Deep Learning	**F1-score**: 0.852 and 0.939			
Scholz et al. (44)	2015	0.83	Surveillance	Simulating the spread of syphilis	SILAS Model	–	–	–	–
Ruan et al. (45)	2021	0.66	Surveillance	Estimating life expectancy	NLP	**Loss**: 5.16E-04			
Ou et al. (46)	2020	0.5	Surveillance	Supporting STIs screening	Complex networks	–	–	–	–
Wang et al. (47)	2022	1.0	Diagnosis	Classifying infectious diseases	MIDDM	72.60%	72.60%	89.45%	–
Elder et al. (48)	2021	1.0	Diagnosis	Classifying STIs	Super Learning (ensemble model)	–	–	–	76%
Bao et al. (49)	2021	1.0	Diagnosis	Predicting STIs diagnosis	GBM	77%	81%	–	85.8%
Dexter et al. (50)	2020	1.0	Diagnosis	Classifying STIs	RF	–	91%	89%	99.22%
Mathur et al. (51)	2021	0.83	Diagnosis	Classifying 20 diseases	CNN ensemble	–	–	–	98%
Lu et al. (52)	2019	0.83	Diagnosis	Identifying indicators	Multivariable Logistic Regression	–	–	–	94.1%
King et al. (53)	2018	0.83	Diagnosis	Classifying STIs	Multivariable Logistic Regression	**c-statistic**: 0.703 and 0.676			
SUN WG (54)	2021	0.66	Diagnosis	Classifying syphilitic uveitis	Multinomial Logistic Regression	100%	–	–	–
Pinoliad et al. (55)	2020	0.66	Diagnosis	Classifying syphilis and other STIs	Deep Learning	90%	100%	58%	–
Pinto et al. (56)	2022	1.0	Health policies	Impact evaluation of health policies	Segmented Linear Regression	–	–	–	–

Boosted GLM, Boosted Generalized Linear Model; STDs, Sexually Transmitted Diseases; ARIMA, Autoregressive Integrated Moving Average; ARIMAX, ARIMA with Explanatory Variable; RMSE, Root Mean Square Error; MAPE, Mean Absolute Percentage Error; LMM, Linear Mixed-effects Model; LASSO, Least Absolute Shrinkage Selection Operator; GEE, Generalized Estimating Equations; CISS+, Chronic Illness Surveillance System - expanded version; NLP, Natural Language Processing; APE, Absolute Percentage Error; KER, Knowledge Embedding Representation; AUPRC, Precision-recall curves; SILAS, Sexual Infections as Large-Scale Agent-based Simulation; STIs, Sexually Transmitted Infections; MIDDM, Multiple Infectious Disease Diagnostic Model; GBM, Gradient Boosting Machine; RF, Random Forest; CNN, Convolutional Neural Network; SUN WG, The Standardization of Uveitis Nomenclature Working Group. *Congenital syphilis.

## 3. Results

The quantitative results of the execution of the SLR protocol are presented in Figure 1. After identification and screening, 26 primary studies were selected as eligible and included in this SLR to respond to the RQ (Table 1). Relevant data were extracted from the eligible studies and described in Table 4.

### 3.1. Research question 01

Different computational approaches applied to syphilis and other STIs were identified in the primary studies. It was observed that mostly and regardless of the context and purpose of the application, primary studies explored different computational models of supervised ML–that is, algorithmic models based on previously labeled data to perform classification or regression tasks.

Data-based computational applications for classification or regression tasks generally involve well-organized and pervasive processes that form the following workflow (57): (i) data acquisition, which will serve as input for computational models after the second stage; (ii) data processing, which prepares the data through denoising, feature extraction, feature selection, and data balancing; (iii) training, testing, and selection of the best computational model for application. In the set of primary studies, a more significant effort was evident in processes (ii) and, mainly, (iii).

With data from electronic records from health centers, and especially considering stage (iii), Xu et al. (31) and Elder et al. (48) proposed the most significant number of computational models applied to the context of syphilis. They used different predictive methods: symbolic; probabilistic; distance-based; margin maximization; connectionists; and ensemble learning. Both articles proposed, respectively, 17 and 16 ML models based on regression algorithms (linear and non-linear), Support Vector Machine (SVM), Bagging Ensemble, Boosting Ensemble, Stacking Ensemble, Random Forest (RF), Naïve Bayes (NB), K-Nearest Neighbor (KNN), Neural Net, and multi-layer perceptron (MLP). As a result, the Boosted Generalized Linear Model (AUC = 0.76) (31) and the Super Learning (cross-validated AUC = 0.76) (48) obtained the best performances.

As for predictive models of ML based on regression and regression for classification, which are widely used in primary studies, the following algorithms were observed: Multivariable/Multivariate Logistic Regression (31, 49, 52, 53); Multinomial Logistic Regression (54); Elastic-Net Regression (31); Logistic Regression (32, 34) Segmented Linear Regression (56); Bayesian Additive Regression Trees (BART) (48); Least Absolute Shrinkage and Selection Operator (LASSO) (35, 48); RIDGE Regression (48); Poisson Regression (36); Generalized Linear Model Logistic Regression (GLM) (48); Boosted GLM (31); Linear Mixed-effects Model (LMM) (35); Linear Regression (32), and Polynomial Regression (32).

Other computational approaches include models based on Deep Learning (43, 49, 55), Convolutional Neural Network Ensemble (51), Decision Tree (47), RF (31, 49, 50), Gradient Boosting Machine (49), Extreme Gradient Boosting (XGBoost) (47–49), Autoregressive Integrated Moving Average (ARIMA) (33, 38, 41), ARIMA with Explanatory Variable (38), Decomposition (38), Generalized Estimating Equations (36), NLP (37, 45), Ontology (42), Complex Networks (46), Knowledge Embedding Representation (40), Sexual Infections as Large-Scale Agent-based Simulation model (44), and Gray Model (39). Table 4 shows the techniques that obtained the best performances in each study and their respective values according to the metric used for evaluation.

### 3.2. Research question 02

The primary included studies show and explore various applications of computational methods in the context of the syphilis. Two large groups of applications stood out: first, in the classification and identification syphilis indicators (47–55); second, in the prediction of STI-related risks, including syphilis (31–36). Both groups employed trained computational models that have learned patterns from a previously known syphilis-related dataset. Such models were able to use those patterns to make predictions or classify new patient data for establishing syphilis diagnosis.

Other scholars, such as Macedo et al. (37), have explored alternative applications and proposed a health surveillance software architecture modeled with ML algorithms and NLP techniques. These techniques can provide preventive recommendations based on specific terms associated with the disease and published scientific articles. Ruan et al. (45), also using NLP, developed a method to estimate health-adjusted life expectancy in China. Zhang et al. (38), Joshi et al. (41), and Scholz et al. (44) developed applications to estimate syphilis incidence in China, estimate syphilis cases in New York State, and simulate a spread of syphilis in the population of Germany, respectively.

Further, by expanding the possibilities of applications based on computational methods in the syphilis context, the studies also presented models built to analyze networks or social media. The goal aimed to interpret and elucidate the relationships of individuals who post about STIs (42) and to forecast outbreaks based on publications and situational awareness by analyzing scientific articles (43). Tissot et al. (40) presented a model for risk assessment of miscarriage during the early stages of pregnancy. In the same perspective of preventive care, Ou et al. (46) proposed an application to help health agents in the STI screening process.

Two studies explored applications for impact analysis. First, Yan et al. (39) used a computational model developed to analyze the impact of the COVID-19 pandemic on the epidemiological changes of STIs in China. In another approach, Pinto et al. (56) evaluated, through an algorithmic model, the effectiveness of public policy actions in Brazil to reduce AS, SIP, and CS rates.

### 3.3. Research question 03

**Figure 2:**
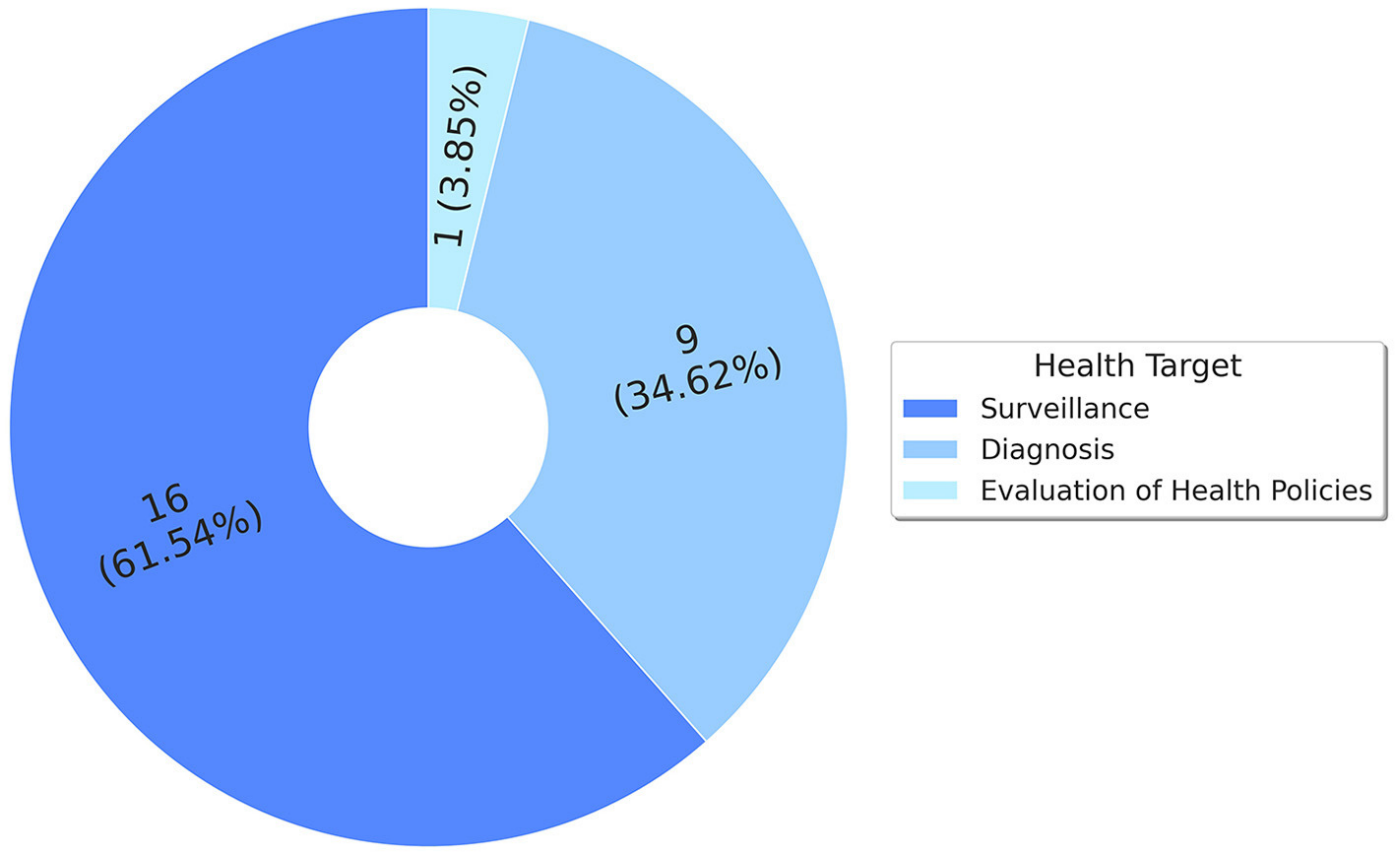
Summary of occurrence of articles by application area.

Considering the perspective of the large area of health sciences, the applications proposed in the included studies focus on seeking and investigating computational solutions within the scope of three subareas (Figure 2). (1) surveillance accounted for 16 studies (61.54%) (31–46); (2) diagnosis for nine related studies (34.62%) (47–55); and (3) evaluation of health policies for only one (3.85%) (56).

For (1) surveillance, studies (31–46) point to promising technologies or computational methods (Sections 3.1 and 3.2) that act as instruments to subsidize and provide technical support to actions mainly related to the epidemiological surveillance of syphilis and other STIs. Not diverging from the purpose of the different subareas, subarea (2) stood out, where studies focus on seeking innovative and scalable solutions for diagnosing syphilis and other STIs (47–55). Regarding (3) evaluation of health policies, Pinto et al. (56) present a computational solution to investigate and statistically measure the effectiveness of strategies and public policy actions which, from the perspective of public health management, is a vital resource to assist and guide decision-making.

## 4. Discussion

Although completely curable, syphilis is a sexually transmitted infection caused by *T. Pallidum*, which is responsible for a silent epidemic wave worldwide (3, 7–9, 58, 59). Even though it is relatively easy to diagnose syphilis through routine laboratory methods, the tests available around the world still present problems, mainly because the most qualified tests are difficult to access, especially in poorer countries. Therefore, the application of computational methods can contribute to the development of new, more accessible (point of care), cheaper, and more accurate tests for the diagnosis of syphilis (60).

Previous studies analyzed the sensitivity and specificity of Syphilis Health Check, a rapid qualitative test to detect human antibodies to *T. Pallidum* (61), or explored the prevalence of syphilis in men who have sex with men (MSM), identifying critical geographic mapping, trends, and data gaps in Latin America and the Caribbean (62). However, in the current paper, we present a systematic review that investigates the application of computational methods as technological tools to support and induce strategies in the context of syphilis. The analysis revealed a diverse set of studies that used computational methods for epidemiological surveillance of syphilis, diagnosis of syphilis, and assessing the impact of public policies.

In this sense, our review shows the utility of computational tools in furthering the general understanding of syphilis which is worsening a global problem, to guide policy and practice to target better public health interventions such as surveillance and prevention, health care service delivery, and the optimal use of diagnostic tools. For instance, Joshi et al. (41) utilized an ARIMA model to investigate the impact of the COVID-19 pandemic on the diagnosis and reporting of STIs, aiming to inform sexual health program planning. The study analyzed New York State STI surveillance data from January 2015 to December 2019 and found that stay-at-home orders contributed to a decline in sexual activity with casual partners, and adversely affected sexual health services, including a reduction in access to diagnostic testing for STIs.

Zhang et al. (38) showed that disease surveillance data could be used to understand syphilis behavior over time using a time-series models. The study revealed a long-term seasonal and increasing trend for the infection, with secondary syphilis showing more significant seasonal fluctuation than other types of the disease. They concluded that patient's likelihood of seeking treatment for secondary syphilis, which is more severe than the other types, was one reason to explain the observed seasonality. Using logistic regression models, Cuffe et al. (34) revealed that several risk factors were associated with a CS case. This finding may potentially support epidemiological surveillance and healthcare services in directing prevention efforts for CS.

Bao et al. (49) demonstrated that it is possible to use ML techniques to predict syphilis infection using datasets that should be available in most settings, such as STIs symptoms, previous syphilis infection, length of residence in the current place, frequency of condom use with casual male sex partners during receptive anal sex, and the number of sex partners.

Dexter et al. (50) alerted to the limitations of predictive models, especially regarding the low generalization power using health data. They cautioned on generalizing the model's performance in the test and validation dataset to general population use. Understanding the descriptors and how to render the model with high generalizability in the test and validation datasets allows the development of reliable models that reach a favorable result within the scope for which it was intended. Algorithmic bias is an important consideration when applying algorithms generated using learning sets and restricted data, as they can further reinforce and augment prevailing inequalities in health systems (63).

There is a need for establishing population-level integrated data sets that are representative, inclusive, and incorporate public health and surveillance data with health service delivery and socio-economic data to improve the utility of AI and ML techniques to strengthen health systems in general and to improve control of syphilis (64, 65). For this disease, there is encouraging development of technological platforms aimed to minimize errors generated by the fragmentation of data used to survey, diagnose and treat syphilis. For example, in Brazil, the Salus Platform Integrates surveillance data with primary health care data and applies ML to improve work processes and response in health crisis scenarios (66, 67). This Platform has also integrated a model of Research on Knowledge, Attitudes, and Practices in the Population into its technological architecture, adapted from the national survey carried out by the Ministry of Health, the Search of Knowledge, Attitudes, and Practices in the Brazilian population (PCAP) (68). With this, it is possible to investigate patient's knowledge, attitudes, and practices related to syphilis, HIV, and other STIs infection.

There are great possibilities with ML to improve and better target surveillance and testing for syphilis and to help inform the development of more efficient and timely diagnostic processes for syphilis and in health surveillance. These developments can help benefit the fight against syphilis, but also other infectious diseases by paving the way for the development of rapid incidence assays to characterize emerging and worsening epidemics (69).

In the context of Brazil, which has the Brazilian National Health System (SUS), with a tripartite governance framework underpinned by a regionalized and hierarchical network of healthcare providers organized according to the complexity of care, behavioral surveys can be carried out when patients seek health care (70, 71). However, for this to happen in SUS, health policies, public health, surveillance, and healthcare service delivery activities need to operate more effectively in an integrated manner (72). Brazil's suboptimal response to COVID-19 has shown the need for better coordination of health policies, public and healthcare delivery, and integrated datasets that can be harnessed for the application of ML methods (73–75).

Results of this study show that analysis using computational techniques could help inform public health and healthcare delivery responses to the worsening syphilis epidemic around the world. But for this to happen, surveillance and policies developed to inform public health and healthcare delivery interventions must be better coordinated. The fact is that health sciences have advanced a lot, particularly with digital health, surpassing the analog world. Therefore, we come from a place where health was more restricted in terms of access to care, as diagnosis methods were only carried out using expensive and difficult-to-access equipment that required super specialists to operate and issue medical reports.

Surveillance actions for STIs such as syphilis, coupled with novel AI-based technologies and tools, contribute toward overcoming the delays in reports drawn on case notification and the shortcomings in current STI data collection. Optimal STI surveillance is contingent on timely and accurate data, yet surveillance data are generally delayed or unavailable (76). In Brazil, which has experienced a syphilis epidemic since 2016 (77), epidemiological reports on syphilis have usually been released belatedly, usually by more than a year. Thus, in this case, how to make decisions that rely only on delayed data that reflects previous scenarios? (78).

Against this background, IA may enhance surveillance, serving as a tool to support decisions about public health interventions in the context of STIs. Therefore, this could provide part of the answer to this public health problem. According to Young et al. (76), available research on STIs has shown that AI can predict syphilis rates at the small-town level by parsing publicly available social media data regarding people's sexual attitudes and behaviors associated with syphilis. This method, known as Rumor analysis, is highly cumbersome through traditional surveillance methods. However, this is not the case when AI-based tools are used because they allow the same analyses to be performed within seconds (79).

Today we are living the transition from this analogical world of health to a fully digital world; the world is experiencing an important process of digital transformation in health. However, for this movement in digital health to be successful and achieve better social results, science must also look at neglected diseases such as syphilis. Advances in health with AI cannot only be used to increase the profits of the health industry; they must also target social inequities and injustices and develop new diagnostic methods to increase access to health for all who need it. This is the way of the future. Using digital health, based on computational methods such as AI, and all its potential to create new diagnostic methods, new tests, and new forms of prevention against STIs, for example, would be a great advance.

Cheaper technologies at the point-of-care that can be operated at distances—telemedicine and telediagnosis—will certainly contribute to reducing inequalities health access, an important contribution to global health (60). Syphilis is a secular disease, but there are indications it is an ancient ailment, rendering senseless the fact it is still a neglected disease by global science. It is necessary to move forward in the present—right now—so that in the future there will be no more children dying from congenital syphilis. This is a very noble goal for science, for health, for digital health and for those who study the application of AI in health.

## 5. Conclusions

This article investigates the literature, based on a systematic review protocol, to identify and highlight studies exploring applications based on computational methods or approaches in the context of syphilis. The execution of the SLR protocol yielded 26 primary studies, which were considered eligible. Our findings reveal a substantial diversification of algorithmic models, regardless of the purpose of application, and three subareas of concentration in the field of health sciences: (1) surveillance (16 studies—61.54%) (31–46); (2) diagnosis (nine studies—34.62%) (47–55); and (3) evaluation of health policies (one study—3.85%) (56).

By showing computational models capable of being tools to support STI control and surveillance actions, the studies show promising outcomes. The use of several ML models in the context of syphilis, for example, exhibit a tendency toward consolidation of algorithms for classification and regression tasks. However, there are still ambitious challenges to be explored, such as evaluating the generalization capacity of models considering different global populations, identifying biases in data, and investigating universal access to applications.

A limitation of this review was the impossibility of defining the best predictors for the analysis of syphilis due to the diversity of methods, datasets, and variables used. In addition, the review findings could not establish a technique with good generalizability for the implemented models.

## Data availability statement

The original contributions presented in the study are included in the article/supplementary material, further inquiries can be directed to the corresponding author.

## Author contributions

GA, FF, and RV contributed to conception and design of the study. GA and FF did collection, organizing, and review of the literature. GA, DB, and FF wrote the first draft of the manuscript. GA, FF, DB, IB, LG-L, AS-M, TP, and RV wrote sections of the manuscript. All authors contributed to manuscript revision, read, and approved the submitted version.
